# Maternal satisfaction with a novel filtered-sunlight phototherapy for newborn jaundice in Southwest Nigeria

**DOI:** 10.1186/1471-2431-14-180

**Published:** 2014-07-10

**Authors:** Bolajoko O Olusanya, Zainab O Imam, Cecilia A Mabogunje, Abieyuwa A Emokpae, Tina M Slusher

**Affiliations:** 1 Centre for Healthy Start Initiative, 286A Corporation Drive, Dolphin Estate Ikoyi, Lagos, Nigeria; 2 Massey Street Children’s Hospital, Lagos, Nigeria; 3 Department of Pediatrics, University of Minnesota and Hennepin County Medical Center, Minneapolis, MN 55415, USA

**Keywords:** Neonatal jaundice, Newborn care, Sunlight exposure, Patient satisfaction, Phototherapy, Developing country

## Abstract

**Background:**

In many resource-limited settings, the availability of effective phototherapy for jaundiced infants is frequently hampered by lack of, or inadequate resources to acquire and maintain conventional electric-powered phototherapy devices. This study set out to ascertain maternal experience and satisfaction with a novel treatment of infants with significant hyperbilirubinemia using filtered sunlight phototherapy (FSPT) in a tropical setting with irregular access to effective conventional phototherapy.

**Methods:**

A cross-sectional satisfaction survey was conducted among mothers of jaundiced infants treated with FSPT in an inner-city maternity hospital in Lagos, Nigeria from November 2013 to March 2014. Mothers’ experience during treatment was elicited with a pretested questionnaire consisting of closed and open-ended items. Satisfaction was rated on a five-point Likert scale. Correlates of overall maternal satisfaction were explored with descriptive and inferential non-parametric statistics.

**Results:**

A total of 191 mothers were surveyed, 77 (40%) of whom had no prior knowledge of neonatal jaundice. Maternal satisfaction was highest for quality of nursing care received (mean: 4.72 ± 0.55, median: 5[IQR: 5–5]) and lowest for physical state of the test environment (mean: 3.85 ± 0.74, median: 4[IQR: 3–4]). The overall rating (mean: 4.17 ± 0.58, median: 4[IQR: 4–5]) and the observed effect of FSPT on the babies (mean: 4.34 ± 0.58, 4[IQR: 4–5]) were quite satisfactory. FSPT experience was significantly correlated with the adequacy of information received (p < 0.0005), test environment (p = 0.002) and the observed effect of FSPT on the child (p < 0.0005). Almost all mothers (98.4%) indicated willingness to use FSPT in future or recommend it to others, although some (30 or 15.7%) disliked the idea of exposing newborns to sunlight.

**Conclusions:**

Mothers of jaundiced newborns in this population are likely to be satisfied with FSPT where it is inevitable as an alternative to conventional electric-powered phototherapy. Adequate information, good test environment and friendly nursing care must be ensured for satisfactory maternal experience.

## Background

Severe neonatal jaundice or hyperbilirubinemia (NNJ) resulting from unconjugated high bilirubin levels is a leading cause for neonatal hospitalization worldwide [1,2]. Phototherapy with electric generated blue-light or light-emitting diode (LED) devices is the treatment of choice, failing which exchange transfusion becomes necessary to avert bilirubin-induced morbidity, mortality, or neurologic dysfunction such as acute bilirubin encephalopathy and kernicterus [3,4]. In many resource-limited settings, the availability of effective phototherapy for jaundiced infants is frequently hampered by lack of or inadequate resources to acquire and maintain conventional phototherapy devices powered by electricity [5,6]. As a result, excessive rates of exchange transfusion are commonly reported along with high incidence of kernicterus and the associated adverse effects [7].

Several studies especially in developing countries in the tropics have reported a common practice of exposing jaundiced infants to direct sunlight as a form of treatment despite concerns about the potential dangers from infrared and ultraviolet rays and sunburn [8-12]. The lack of viable and low-cost alternatives to conventional phototherapy has, therefore, prompted the development of specially filtered sunlight phototherapy (FSPT) canopies using commercially available window-tinting films to provide protection from infrared and ultraviolet rays [13]. The safety and efficacy of this novel treatment have been demonstrated in Nigerian newborns [14]. Preliminary findings from a randomized controlled trial in the same population also suggests that FSPT is no less efficacious than conventional phototherapy devices [15].

Patient satisfaction is an important and widely accepted component of effective health care delivery worldwide [16]. This is because the involvement of the users of clinical health services facilitates improved outcomes from satisfied patients through improved compliance and continuity of care. The acceptability of FSPT among mothers has not yet been investigated. Such evidence is essential for the effective use of this novel treatment as an alternative to the prevailing cultural practice of exposing jaundiced infants to direct sunlight or use of other potentially harmful traditional therapies. This study, therefore, set out assess maternal satisfaction with FSPT for babies with mild to moderate hyperbilirubinemia (with total serum bilirubin levels typically below 12 mg/dL or 205 μmol/L) at its pilot site.

## Methods

**Figure 1:**
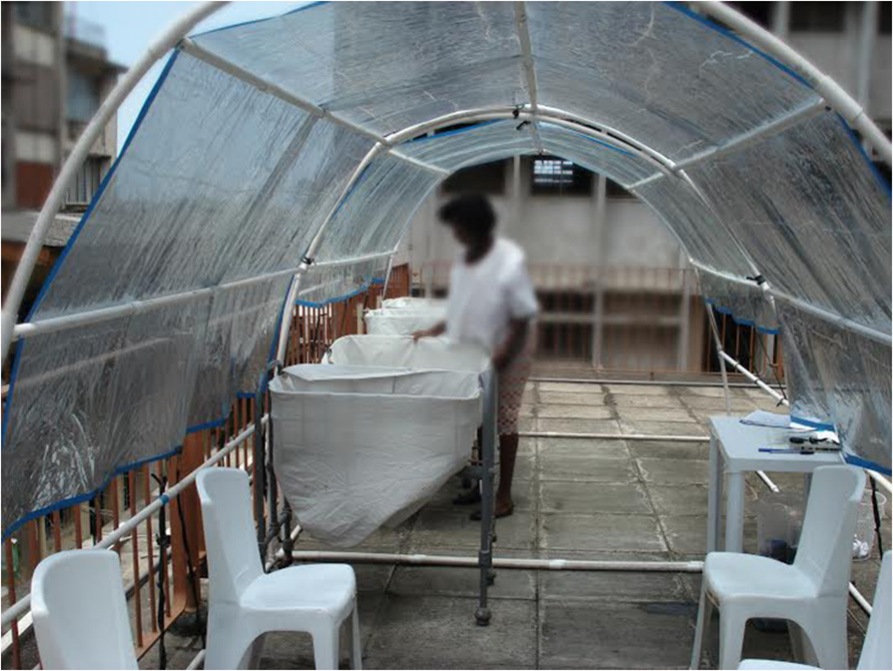
A typical canopy for filtered sunlight phototherapy with seating capacity for four mother-infant pairs and two nursing staff.

This cross-sectional survey was conducted at the Island Maternity Hospital (IMH) in Lagos, Nigeria among consenting mothers whose newborns were treated for jaundice using FSPT between November 2013 and April 2014. IMH is a public health institution owned and managed by the Lagos State Government. It is the oldest maternity hospital in Nigeria providing specialist services to several private and public hospitals within metropolitan Lagos. The newborn unit in IMH is managed exclusively by a team of pediatricians drawn from a nearby children’s hospital also owned by the state government. The study was conducted according to the guidelines laid down in the Declaration of Helsinki, and ethical approval for all procedures involving human subjects were approved by the Lagos State Government Health Service Commission (Ref: SHMB/728/VOL. VII/962). As a requirement for obtaining informed consent under the institutional ethical approval for the substantive study, mothers were given a standardized and documented package of information on the FSPT and provided with opportunity to seek clarification on any aspects [14]. Information on the significance of jaundice in newborns, purpose of the intervention, description of FSPT, the procedures and requirements for tests, potential risks such as dehydration, hypothermia, hyperthermia and sunburn were included. Mothers were assured of frequent monitoring by a dedicated health worker and the confidentiality of all personal information. It was also emphasized that their participation was optional and could be withdrawn at any stage of the study at their request. FSPT was delivered to eligible infants through a custom-made canopy covered with pre-tested (*in vitro* and *in vivo*) window tinting films as previously described (see Figure 1) [14,15]. The films were duly approved by the National Agency for Food and Drug Administration and Control of Nigeria. This intervention was offered at no charge to parents as part of the package of newborn care in this publicly-funded hospital.

The survey instrument (Additional file 1) was adapted from a questionnaire on maternal knowledge, attitude and practice regarding neonatal jaundice that has been successfully implemented in three cities in Nigeria [17]. The three-part questionnaire was administered by a trained research worker not directly involved with clinical management under the pilot studies [14,15]. The first part of the questionnaire included socio-demographic data of respondents such as maternal age, marital status, ethnicity, religion, number of children as well as self and spouse’s educational status. The next part mainly consisted of seven Likert-type closed-ended questions requiring the respondents to rank their experience with FSPT as ‘Very Good’ , ‘Good’ , ‘Fair’ , ‘Poor’ and ‘Very Poor’ , scored as 5 to 1 respectively. The third part was predominantly open-ended seeking to establish the respondent’s prior experience with an infant with jaundice and the actions taken compared to FSPT. It also elicited the respondent’s likes or dislikes about FSPT as well as disposition to future use or recommendation of FSPT. The overall study design was guided by relevant recommendations for patient satisfaction surveys in general [18,19].

The required sample size was calculated using single population proportion formula (n = (Z 1-α/2)2 p (1-p)/ d2) with the following assumptions: expected proportion (p) of the study participants who were satisfied with FSPT (75%), marginal error (d) 5% and confidence interval of 95%. We postulated that 75% of mothers whose infants were treated over a two-year period will be satisfied based on evidence from local studies reporting sunlight exposure of jaundiced infants as a general practice [11,12]. The minimum sample size was computed as 183, after allowance for a 5% non-participation rate.

The quantitative data was analyzed with descriptive and nonparametric inferential statistics using IBM SPSS Statistics for Windows, Version 20.0 (Armonk, NY: IBM Corp) software. The socio-demographic characteristics of the respondents sub-divided into those with or without prior knowledge of neonatal jaundice were summarized in a frequency table. The mean (± standard deviation) and median (plus interquartile range) scores for the Likert-type items were computed to rank the satisfaction levels for the seven dimensions. The correlation between these seven dimensions was assessed with Spearman’s rho coefficients. Differences in satisfaction levels across selected demographic variables (ethnicity, religion, maternal and paternal education, number of children and prior knowledge of neonatal jaundice) were evaluated with Kruskal-Wallis or Mann–Whitney U- test as appropriate because the numeric dependent variables were non-Gaussian. P-values less than or equal to 0.05 were considered statistically significant. The specific responses to the open-ended questions on the mothers’ likes and dislikes about FSPT were analyzed with ATLASti 7.1.8 qualitative data analysis software coded into common themes such as patient information on FSPT, nature of treatment, test environment and quality of nursing care. Mothers with blank and non-specific responses were excluded. The overall reporting was guided by the STROBE checklist (Additional file 2).

## Results

A total of 191 consenting mothers, comprising 77 (40.0%) without, and 114 (60.)% with prior knowledge of neonatal jaundice were enrolled for the study. All but two of the mothers with prior knowledge of jaundice cited health workers or antenatal clinics as their sources of information. No eligible mother declined participation in the survey. A demographic overview of the participants based on prior knowledge of neonatal jaundice is presented in Table 1. No teenage mother was enrolled for the study, and only 4 (2.1%) were older than 35 years. The majority of the mothers were married (98.4%), belonged to the Yoruba tribe (76.4%) and were self-employed (61.8%). About half (51.3%/51.4%) of the mothers and their spouses had post-secondary education. A high proportion (47.1%) of the mothers were primiparous and slightly over half (55%) were Muslims. There were virtually no differences between the two groups of mothers except that mothers with post-secondary education were significantly more likely to have a prior knowledge of neonatal jaundice compared to mothers with lesser or no education (χ^2^ = 7.87, p = 0.005). Maternal experiences with key aspects of FSPT are summarized in Table 2. Maternal satisfaction was highest for quality of nursing care received (mean: 4.72 ± 0.55, median: 5 [IQR: 5–5]) and lowest for physical state of the test environment (mean: 3.85 ± 0.74, median: 4 [IQR: 3–4]). The overall experience was more than average (mean: 4.17 ± 0.58, median: 4 [IQR: 4–5]), as well as the observed effect of FSPT on the babies (mean: 4.34 ± 0.58, 4 [IQR: 4–5]). The adequacy of information provided on FSPT, opportunity to feed without taking their child out of phototherapy as would be required in conventional phototherapy, and the opportunity to socialize with other mothers were also rated as satisfactory The Spearman’s correlation analysis shows that the overall FSPT experience was significantly but modestly correlated with the adequacy of information received (r = 0.253, p < 0.0005), test environment (r = 0.223, p = 0.002) and the observed effect of FSPT on the child (r = 0.419, p < 0.0005) (Table 3). The satisfactory rating on quality of nursing care was also significantly but modestly correlated with adequacy of information received (r = 0.203, p = 0.005) and the opportunity to socialize with other mothers (r = 0.213, p = 0.003). The opportunity to feed and bond with the child while receiving treatment was significant and highly correlated with the opportunity to socialize with other mothers (r = 0.519, p < 0.0005). The observed effect of FSPT was also significantly but modestly related to the educational information received by the mothers (r = 0.292, p < 0.0005).

**Table 1:** Characteristics of participating mothers in the satisfaction survey on FSPT (n = 191)

**Factors**	**Not previously heard of NNJ n = 77 (%)**	**Previously heard of NNJ n = 114 (%)**	**Total N = 191 (%)**	**p-value**
Maternal age (Years)				0.528
< 20	0 (0.0)	0 (0.0)	0 (0.0)	
20 – 35	76 (98.7)	111 (97.4)	187 (97.9)	
>35	1 (1.3)	3 (2.6)	4 (2.1)	
Marital status				0.804
Single	1 (1.3)	2 (1.8)	3 (1.6)	
Married	76 (98.7)	112 (98.2)	188 (98.4)	
Number of living children				0.933
1	36 (46.8)	54 (47.4)	90 (47.1)	
2 or more	41 (53.2)	60 (52.6)	101 (52.9)	
Ethnicity				0.514
Hausa	5 (6.5)	4 (3.5)	9 (4.7)	
Ibo	10 (13.0)	10 (8.8)	20 (10.5)	
Yoruba	57 (74.0)	89 (78.1)	146 (76.4)	
Others	5 (6.5)	11 (9.6)	16 (8.4)	
Religion				0.620
Christianity	33 (42.9)	53 (46.5)	86 (45.0)	
Islam	44 (57.1)	61 (53.5)	105 (55.0)	
Education				0.005
None/Primary	6 (7.8)	11 (9.6)	17 (8.9)	
Secondary	41 (53.2)	35 (30.7)	76 (39.8)	
Technical	11 (14.3)	23 (20.2)	34 (17.8)	
Tertiary	19 (24.7)	45 (39.5)	64 (33.5)	
Education of spouse				0.425
None/Primary	1 (1.3)	3 (2.6)	4 (2.1)	
Secondary	33 (42.9)	37 (32.5)	70 (36.5)	
Technical	11 (14.3)	15 (13.2)	26 (13.8)	
Tertiary	32 (41.6)	59 (51.8)	91 (47.6)	
Occupation				0.755
Unemployed	11 (14.3)	18 (15.8)	29 (15.2)	
Self-employed	50 (64.9)	68 (59.6)	118 (61.8)	
Formal job	16 (20.8)	28 (24.6)	44 (23.0)	
Occupation of spouse				0.503
Unemployed	0 (0.0)	2 (1.8)	2 (1.0)	
Self-employed	40 (51.9)	59 (51.8)	99 (51.8)	
Formal job	37 (48.1)	53 (46.5)	90 (47.1)	

FSPT: Filtered sunlight phototherapy.

**Table 2:** Summary of ratings for key aspects maternal experience with FSPT

	**Dimensions of satisfaction**	**Mean score**	**SD**	**Median score**	**IQR**
1.	Adequacy of information received on FSPT before treatment	4.36	0.54	4.0	4 - 5
2.	Quality of nursing care received during treatment	4.72	0.55	5.0	5 - 5
3.	Physical state of treatment environment	3.85	0.74	4.0	3 - 4
4.	Opportunity to feed and bond with baby while receiving FSPT	4.41	0.61	4.0	4 - 5
5.	Opportunity to socialize with other mothers during treatment	4.34	0.54	4.0	4 - 5
6.	Observed effect of FSPT in the baby	4.34	0.58	4.0	4 - 5
7.	Overall experience with FSPT	4.17	0.58	4.0	4 - 5

FSPT: Filtered sunlight phototherapy, SD: Standard deviation, IQR: Inter-quartile range.

**Table 3:** Summary of Spearman’s correlation analysis of dimensions of satisfaction with FSPT

		**Information received**	**Nursing care quality**	**Treatment environment**	**Feeding & bonding**	**Socialization**	**Observed FSPT effect**	**Overall FSPT experience**
Information received	Rho-Coefficient	1.000						
	Sig. (2-tailed)	.						
Nursing care quality	Rho-Coefficient	.203^**^	1.000					
	Sig. (2-tailed)	.005	.					
Treatment environment	Rho-Coefficient	.109	-.084	1.000				
	Sig. (2-tailed)	.135	.249	.				
Feeding & bonding	Rho-Coefficient	.322^**^	.185^*^	.228^**^	1.000			
	Sig. (2-tailed)	.000	.011	.002	.			
Socialization	Rho-Coefficient	.370^**^	.213^**^	.087	.519^**^	1.000		
	Sig. (2-tailed)	.000	.003	.234	.000	.		
Observed FSPT effect	Rho-Coefficient	.292^**^	-.021	.071	.152^*^	.075	1.000	
	Sig. (2-tailed)	.000	.770	.334	.037	.304	.	
Overall FSPT experience	Rho-Coefficient	.253^**^	-.099	.223^**^	.145^*^	.115	.419^**^	1.000
	Sig. (2-tailed)	.000	.174	.002	.045	.114	.000	.

N = 191; **Correlation is significant at the 0.01 level (2-tailed). *Correlation is significant at the 0.05 level (2-tailed). FSPT: Filtered sunlight phototherapy.

The overall experience with FSPT did not differ significantly among the mothers based on their demographics except for the number children (Table 4). The multiparous mothers had a higher level of satisfaction than the first-time mothers (p = 0.035) while Christian mothers were significantly more satisfied with the quality of nursing care than Muslim mothers (p = 0.014). Mothers whose spouses had post-secondary education were significantly less satisfied with the test environment than those with lesser or no education (p = 0.031), while mothers (p = 0.019) and their spouses (p = 0.001) without post-secondary education were significantly more satisfied for the opportunity to socialize with other mothers than couples with post-secondary education. Satisfaction with the educational information, opportunity to feed and bond with the child, as well as the observed positive effect of FSPT, were independent of the mothers’ demographic profile.

**Table 4:** Differences in satisfaction levels across demographic status of respondents based on the Kruskal-Wallis or Mann–Whitney test

**Profile**	**Overall FSPT experience χ**^**2 **^**(p-value)**	**Information received χ**^**2 **^**(p-value)**	**Nursing care quality χ**^**2 **^**(p-value)**	**Treatment environment χ**^**2 **^**(p-value)**	**Feeding & bonding χ**^**2 **^**(p-value)**	**Socialization χ**^**2 **^**(p-value)**	**Observed FSPT effect χ**^**2 **^**(p-value)**
Ethnicity	0.734 (0.865)	1.125 (0.771)	1.135 (0.765)	2.495 (0.476)	1.300 (0.729)	1.614 (0.656)	0.336 (0.953)
Religion^a^	1.698 (0.193)	0.364 (0.546)	**5.963 (0.014)**	2.080 (0.149)	0.035 (0.851)	0.003 (0.957)	0.003 (0.955)
Maternal education^b^	0.555 (0.456)	0.110 (0.740)	0.777 (0.378)	3.178 (0.075)	0.005 (0.942)	**5.524 (0.019)**	0.193 (0.660)
Spouse’s education^c^	0.004 (0.951)	0.488 (0.485)	0.028 (0.867)	**4.663 (0.031)**	0.208 (0.648)	**11.332 (0.001)**	0.057 (0.811)
Number of children^d^	**4.432 (0.035)**	0.830 (0.362)	0.025 (0.875)	0.754 (0.385)	0.350 (0.554)	3.089 (0.079)	0.004 (0.950)
Ever heard of NNJ	0.090 (0.764)	1.040 (0.308)	2.999 (0.083)	2.587 (0.108)	0.051 (0.821)	0.437 (0.509)	3.402 (0.065)

^a^Christians compared to Muslims; ^b,c^Secondary/Primary compared to Postsecondary; ^d^Multiparous compared to primiparous. FSPT: Filtered sunlight phototherapy.

The most frequently cited positive attributes of FSPT were the friendliness of the nursing staff and the observed effectiveness of this treatment for neonatal jaundice (Table 5). The most frequently reported negative attributes were the exposure of the newborns to “hot” sun and ambience of the test environment. More of first-time mothers (66.7%) than multiparous mothers (60.9%), those without post-secondary education (76.5%) than those with post-secondary education (56.7%), Muslims (70.4%) than Christians (55.0%) and Yorubas (67.6%) than non-Yorubas (50.0%) complained about the sun exposure of the newborns but the differences between these groups were not statistically significant (data not shown). All but one of the 47 mothers who cited specific dislikes about FSPT indicated that they would in future receive this treatment and recommend it to others. This one mother, a university graduate, disliked the exposure of the newborn to sun but ironically rated her overall experience with FSPT as “good” and the observed effect on the baby as “very good”. Nine mothers reported at least one older child with jaundice, 3 of whom cited exposure to sunlight as treatment given to the child, 1 reported the use of herbal mixture and the rest had hospital treatment. One mother from this group reported that the child finally died while another reported delayed development with impaired hearing. Almost all mothers (98.4%) indicated willingness to use FSPT in future or recommend it to others.

**Table 5:** Summary of themes from open-ended comments on FSPT experience

**Comments**	**N**	**% of N**	**% all Mothers (n = 191)**
**What respondents like about FSPT**	N = 98	N = 98	51.3
• Friendliness of nursing staff	47	48.0	24.6
• Effectiveness of FSPT for treating jaundice	36	36.7	18.8
• Usefulness of information on FSPT	9	9.2	4.7
• Shade from direct sun exposure	4	4.1	2.1
• Eye pad cover for baby	2	2.0	1.0
**What respondents dislike about FSPT**	[N = 47]	[N = 47]	24.6
• Exposure of a newborn to “hot” sun	30	63.8	15.7
• Condition of treatment environment	10	21.3	5.2
• Long duration of treatment	5	10.6	2.6
• Attention to baby’s needs	2	4.3	1.0

FSPT: Filtered sunlight phototherapy.

## Discussion

This study set out to establish the views of mothers on a novel treatment of neonatal jaundice with FSPT in a clinical setting that is frequently constrained to deliver effective conventional blue-light phototherapy. This is against the backdrop of reported concerns regarding the safety of sunlight exposure for therapeutic purposes including the treatment of jaundice [20-22]. In fact, several clinical guidelines for the management of neonatal jaundice expressly prohibit exposure to sunlight as a form of treatment [23-26], perhaps, except in Ghana where the national treatment guidelines for newborn care made a cursory allowance for sunlight exposure of jaundiced infants [27].

The overarching finding in this study is that mothers whose jaundiced infants received FSPT were quite satisfied with this form of treatment despite needing to keep their infants exposed throughout the day. Regardless of the reservations expressed by a few mothers on sunlight exposure, the vast majority expressed willingness to accept this treatment in future, if required, and were confident enough to recommend it to other mothers. As this is the first survey among mothers regarding FSPT, there are no comparable studies yet. However, two studies have explored maternal knowledge, attitude and practice regarding exposure of babies to “unfiltered” sunlight [9,22]. In the first study from Australia among 114 Caucasian women, 36% thought it was a good idea to sun their baby to treat jaundice; 21.1% disagreed and the rest (43%) were unsure [9]. In fact, one-third of the mothers indicated that they would sun their baby with suspected jaundice without recourse to a doctor. About 24% of the mothers had sunned their baby to treat jaundice either through a window, on a veranda or exposed to direct sunlight. Women who had sunned their baby suspected with jaundice were found to be significantly more likely than other women to be in favor of this treatment (p = 0.00001). It was further reported that 41% of the mothers were advised to sun their baby to treat jaundice by a hospital nurse/midwife, 28% by a pediatrician/medical officer and 6% by both physicians and nurses. A second study conducted in Turkey, sought the views of 118 mothers regarding the possible use of sun exposure as treatment for jaundice [22]. About 15 (12.7% of 118 or 14% of the 107 mothers who responded) indicated that sunlight was good for jaundice but there was no information on the number of mothers who had actually used this form of treatment. Of those who responded to this question on the use of sunlight for treating jaundice, 7 (6.5%) mothers did consider this treatment as good while the vast majority (79.4%) had no idea. Besides treatment for neonatal jaundice, sun exposure is also used in these two countries by mothers to alleviate nappy rash, ensure adequate production of vitamin D and for bone development unlike the practice in our study population where sun exposure is predominantly associated with treatment of jaundice.

In settings with good access to functional conventional phototherapy, FSPT would probably be unnecessary. Educational intervention to discourage mothers and health professionals from using unfiltered sunlight treatment for newborns with jaundice in such settings may therefore be justified [20,21]. However, where access to conventional phototherapy is lacking, FSPT needs to be considered for infants at risk of severe jaundice and kernicterus as the benefit is likely to exceed any potential harm. It is important to make a clear and emphatic distinction between untested films or filters and FSPT that pre-tested films that are duly approved by the relevant Safety Regulatory Authorities. Exposure of newborns to direct and unfiltered sunlight should under any circumstances be discouraged because of the potential and invisible harms from ultraviolet radiation and infrared rays. This is because mothers are likely to be dissuaded by the widely publicized therapeutic effect of this age-long practice on the child. As our study would suggest, a high proportion of mothers are likely to learn about neonatal jaundice from health workers usually during antenatal clinics. This forum provides opportunity for proper education on the dangers of indiscriminate exposure of jaundice babies to sunlight.

Where FSPT is contemplated, the ideal sunlight PT film should: (i) block ultraviolet radiation to <1% that of unfiltered sunlight (~2000 μW/cm^2^); (ii) block infrared sufficiently to maintain patient thermostasis; (iii) transmit sufficient level of therapeutic blue light; and (iv) be transparent to facilitate visibility of the patient for purposes of clinical management [13]. The two films used for this study excluded virtually all UVA, UVB and UVC radiation. For instance, the film chosen for use during overcast sky periods, transmitted 79% of the >400–520 nm wavelength blue light and only 0.1% of the 315–400 nm UVA while the film chosen for periods of direct sunlight, transmitted 33% of the 400–520 nm wavelength light and 0.4% of the UVA radiation [13,14]. The two films provided partial shade that reduced the temperature under a cloudy and cloudless sky by 6°C and 9.5°C, respectively. However, studies reporting the use of some form of “filtered” sunlight phototherapy in which the baby is shielded with tinted glass window rarely provide safety data on the level of radiation which makes comparison with our study or an independent assessment difficult [9,28]. One study from Bangladesh, for example, only reported that infants were exposed to the sun for 1-2hours in the early morning and afternoon using a “filter of tinted glass” [28].

The opportunity to feed and bond with child while receiving treatment was appreciated by mothers, unlike when they have to be separated under conventional phototherapy. Our study also highlights other essential components of maternal satisfaction with FSPT such as adequacy of information provided regarding the treatment, the physical environment, quality of nursing care received which would include friendliness of the health workers, and the concept of group FSPT under a single canopy which offered opportunity for socializing with other mothers. The higher level of satisfaction among the multiparous mothers could be a reflection of their experience with newborn care and the fact that this treatment did not require any form of medication, unlike other childhood illnesses. Our study also suggests that mothers who belonged to high social class by virtue of their (and spouse’s) educational status, and who ordinarily would have chosen to deliver in a private hospital, are likely to be more sensitive to the physical ambience of the test environment in this public hospital. Efforts towards improving the ambience of the test environment should also be considered in offering FSPT.

While the safety and efficacy of the FSPT used in this population have been rigorously demonstrated [14], its widespread promotion is still subject to further evaluation especially regarding how to handle interruptions due to inclement weather conditions. The findings in this present study should, therefore, be considered as exploratory, as with most innovative health care interventions. One limitation of this study is that we were not able to determine if maternal satisfaction was correlated with the severity of jaundice and age of the child on enrolment as only infants with mild to moderate jaundice were treated. We could not ascertain maternal views on preterm infants who were jaundiced as they were excluded from FSPT treatment. The psychometric properties of the questionnaire were not statistically evaluated. However, considering that the questionnaire was adapted from a properly validated questionnaire earlier used among a different set of mothers in a multi-centre survey in three distinct geographical regions in the country, there is no reason to doubt its validity in this study population [17]. Moreover, the overall maternal satisfaction ratings were consistent with the expected efficacy of FSPT on the enrolled infants as earlier reported [13,14].

## Conclusion

The use of FSPT for the treatment of infants with mild to moderate jaundice to forestall the risk of severe jaundice and kernicterus is likely to be acceptable to mothers in the absence of conventional phototherapy. Adequate information, good test environment and friendly nursing care must be ensured for satisfactory maternal experience. However, only FSPT with pre-tested and duly approved films should be considered. This study has not provided any evidence in support of indiscriminate exposure of infants with jaundice to sunlight without adequate safety precaution to shield the child from potentially harmful ultraviolet and infrared rays notwithstanding the perceived therapeutic benefit of this treatment. While the potential utility and acceptance of FSPT have been demonstrated in this population, further assessment especially for all categories of infants not enrolled in this study will be needed before this form of treatment can be widely promoted.

## Abbreviations

FSPT: Filtered Sunlight Phototherapy; IMH: Island Maternity Hospital; NNJ: Neonatal Jaundice or Hyperbilirubinemia; LED: Light-emitting diode; IQR: Inter-quartile range; UV (A: B or C), Ultraviolet rays A, B or C.

## Competing interests

The authors declare that they have no competing interests.

## Authors’ contributions

BOO conceived, designed and supervised the study. BOO analyzed the data and drafted the manuscript. CAM, ZOI and AAE participated in the data analysis and interpretation. TMS led the substantive research that formed the basis of the study and made critical intellectual inputs to the final manuscript. All authors read and approved the final manuscript.

## Pre-publication history

The pre-publication history for this paper can be accessed here:

http://www.biomedcentral.com/1471-2431/14/180/prepub

## Supplementary Material

Additional file 1 Questionnaire on maternal experience with FSPT. Click here for file

Additional file 2 **STROBE Checklist.** From: von Elm E, Altman DG, Egger M, Pocock SJ, Gøtzsche PC, et al. (2007) The Strengthening the Reporting of Observational Studies in Epidemiology (STROBE) Statement: Guidelines for Reporting Observational Studies. PLoS Med 4(10): e296. doi:10.1371/journal.pmed.0040296. Click here for file
