# Supplementary material for: Maternal satisfaction with a novel filtered-sunlight phototherapy for newborn jaundice in Southwest Nigeria
Source: BMC Pediatr. 2014 Jul 10;14:180. doi: 10.1186/1471-2431-14-180 (PMC4099408; doi:10.1186/1471-2431-14-180)
Supplement: Additional file 1 — Questionnaire on maternal experience with FSPT. [file 1471-2431-14-180-S1.pdf]

MATERNAL EXPERIENCE WITH FILTERED SUNLIGHT PHOTOTHERAPY

Instruction: All parts (A, B, C & D) of this form must be duly completed by the respondent/interviewer **ID. NO…………**

PART A: RESPONDENT'S PROFILE (Tick or describe as applicable)

1. Age: …. or Group: <20☐ 20-35☐ >35☐Years. **2. Marital Status:** Single☐ Married ☐ Separated/Divorced☐ Widow ☐

3. Ethnicity: Yoruba ☐ Hausa ☐ Ibo ☐ Other………………… **4. Religion:** Christian ☐ Muslim ☐ Other ☐

5. No. of Children: ……….. **6. No. Alive:** ……… **7. Any Twins:** Yes ☐ No ☐ **8. Age of Last Child:** …… (Years)

9. Your Education: None ☐ Primary ☐ Secondary ☐ Technical/Teachers College ☐ University ☐

10. Husband's Education: None ☐ Primary ☐ Secondary ☐ Technical/Teachers College ☐ University ☐

11. Your Occupation: ……………………………………… **12. Husband's Occupation:** ………………………………..

PART B: EXPERIENCE WITH FILTERED SUNLIGHT PHOTOTHERAPY (Tick or describe as applicable)

13. Have you heard about jaundice in babies during antenatal clinic? Yes ☐ **No** ☐

If Yes from what source: Doctor☐ Nurse/Midwife ☐ Friend/Neighbor/Relation ☐Radio/TV ☐ Antenatal Clinic ☐ Other ☐ ……………………………………………………………………………………

14. Have you heard about filtered sunlight phototherapy during antenatal clinic? Yes ☐ No ☐

15. Rate your overall experience with filtered sunlight phototherapy? V. Good ☐ Good ☐ Fair ☐ Poor ☐ V. Poor ☐

16. How will you rate the adequacy of the information given to you? V. Good ☐ Good ☐ Fair ☐ Poor ☐V. Poor☐

17. How will you rate the nursing care? V. Good ☐ Good ☐ Fair☐ Poor ☐ V. Poor ☐

18. How will you rate the treatment environment? V. Good ☐ Good ☐ Fair ☐ Poor ☐ V. Poor ☐

19. How will you rate the opportunity to feed and bond with your child? V. Good ☐ Good ☐ Fair ☐ Poor☐ V. Poor☐

20. How will you rate the opportunity to socialize with other mothers? V. Good ☐ Good ☐ Fair ☐ Poor☐ V. Poor ☐

21. How will you rate the effect of filtered sunlight on your baby? V. Good ☐ Good ☐ Fair ☐ Poor ☐ V. Poor ☐
………

PART C: COMPARING FILTERED SUNLIGHT WITH OTHER TREATMENT MODALITIES (Tick or describe as applicable)

22. Have you had a baby with jaundice? a) Yes ☐ No ☐ b) **One of Twins?** Yes ☐ No ☐

23. If Yes, who first discovered the jaundice? Self ☐ Relation/Neighbour ☐ Doctor/Nurse ☐ Other …………

24. What did you do for the baby? ....

25. What treatment was given to the baby? a)………………………………………………b) **How long?** .... (Days)

26. What was the final outcome? Baby recovered fully ☐ Baby died ☐ Baby recovered with complications* ☐

*Nature of complications: ………………………………………………………………………………

27. How did sunlight phototherapy compare with the previous treatment given to your other baby with jaundice?

……………………………………………………………………………………………………

28. Now focusing on your experience of sunlight phototherapy:

a) What did you not like about the treatment? ………………………………………………………….

b) What did you like? ……………………………………………………………………………………..

29. In future will you receive sunlight phototherapy for another baby? Yes☐ No ☐

30. Will you recommend it to others? Yes ☐ No ☐

PART D: FOR INTERVIEWER ONLY. Use the back of this sheet for any additional respondent's information

Respondent's consent obtained: Yes ☐ No ☐ **Reason for declining**: ……………………………………

Interviewer's Name: ……………………………………………. Status ………………… Date ……………………….

© NEONATAL JAUNDICE STUDY GROUP OF NIGERIA
