# Supplementary material for: Maternal satisfaction with a novel filtered-sunlight phototherapy for newborn jaundice in Southwest Nigeria
Source: BMC Pediatr. 2014 Jul 10;14:180. doi: 10.1186/1471-2431-14-180 (PMC4099408; doi:10.1186/1471-2431-14-180)
Supplement: Additional file 2 — STROBE Checklist. From: von Elm E, Altman DG, Egger M, Pocock SJ, Gøtzsche PC, et al. (2007) The Strengthening the Reporting of Observational Studies in Epidemiology (STROBE) Statement: Guidelines for Reporting Observational Studies. PLoS Med 4(10): e296. doi:10.1371/journal.pmed.0040296. [file 1471-2431-14-180-S2.doc]

**STROBE Statement—Checklist of items included in paper: Olusanya et al 2014.**

|  | Item No | Recommendation |
| --- | --- | --- |
| **Title and abstract** | 1 | (*a*) Indicate the study’s design with a commonly used term in the title or the abstract: **Page 1** |
|  |  | (*b*) Provide in the abstract an informative and balanced summary of what was done and what was found: **Page 1** |
| Introduction | | |
| Background/rationale | 2 | Explain the scientific background and rationale for the investigation being reported: **Pages 1&2** |
| Objectives | 3 | State specific objectives, including any prespecified hypotheses: **Page 2** |
| Methods | | |
| Study design | 4 | Present key elements of study design early in the paper: **Page 2** |
| Setting | 5 | Describe the setting, locations, and relevant dates, including periods of recruitment, exposure, follow-up, and data collection: **Page 2** |
| Participants | 6 | (*a*) Give the eligibility criteria, and the sources and methods of selection of participants:  **Page 2** |
| Variables | 7 | Clearly define all outcomes, exposures, predictors, potential confounders, and effect modifiers. Give diagnostic criteria, if applicable: **Pages 2&3** |
| Data sources/ measurement | 8* | For each variable of interest, give sources of data and details of methods of assessment (measurement). Describe comparability of assessment methods if there is more than one group: **Page 2** |
| Bias | 9 | Describe any efforts to address potential sources of bias: **Page 2** |
| Study size | 10 | Explain how the study size was arrived at: **Page 3** |
| Quantitative variables | 11 | Explain how quantitative variables were handled in the analyses. If applicable, describe which groupings were chosen and why: **Page 3** |
| Statistical methods | 12 | (*a*) Describe all statistical methods, including those used to control for confounding: **Page 3** |
|  |  | (*b*) Describe any methods used to examine subgroups and interactions: **Not applicable (N/A)** |
|  |  | (*c*) Explain how missing data were addressed: **Page 3** |
|  |  | (*d*) If applicable, describe analytical methods taking account of sampling strategy: **Page 3** |
|  |  | (*e*) Describe any sensitivity analyses: **N/A** |
| Results | | |
| Participants | 13* | (a) Report numbers of individuals at each stage of study—eg numbers potentially eligible, examined for eligibility, confirmed eligible, included in the study, completing follow-up, and analysed: **Page 3** |
|  |  | (b) Give reasons for non-participation at each stage: **N/A** |
|  |  | (c) Consider use of a flow diagram: **N/A** |
| Descriptive data | 14* | (a) Give characteristics of study participants (eg demographic, clinical, social) and information on exposures and potential confounders: **Table 1** |
|  |  | (b) Indicate number of participants with missing data for each variable of interest: **Table 1** |
| Outcome data | 15* | Report numbers of outcome events or summary measures: **Tables 1-5** |
| Main results | 16 | (*a*) Give unadjusted estimates and, if applicable, confounder-adjusted estimates and their precision (eg, 95% confidence interval). Make clear which confounders were adjusted for and why they were included: **N/A** |
|  |  | (*b*) Report category boundaries when continuous variables were categorized: **N/A** |
|  |  | (*c*) If relevant, consider translating estimates of relative risk into absolute risk for a meaningful time period: **N/A** |
| Other analyses | 17 | Report other analyses done—eg analyses of subgroups and interactions, and sensitivity analyses: **N/A** |
| Discussion | | |
| Key results | 18 | Summarise key results with reference to study objectives: **Pages 5-7** |
| Limitations | 19 | Discuss limitations of the study, taking into account sources of potential bias or imprecision. Discuss both direction and magnitude of any potential bias. **Page 7** |
| Interpretation | 20 | Give a cautious overall interpretation of results considering objectives, limitations, multiplicity of analyses, results from similar studies, and other relevant evidence: **Page 7** |
| Generalisability | 21 | Discuss the generalisability (external validity) of the study results: **Page 7** |
| Other information | | |
| Funding | 22 | Give the source of funding and the role of the funders for the present study and, if applicable, for the original study on which the present article is based: **Page 7** |

*Give information separately for exposed and unexposed groups.
